# Generation of Chicken IgY against SARS-COV-2 Spike Protein and Epitope Mapping

**DOI:** 10.1155/2020/9465398

**Published:** 2020-10-17

**Authors:** Yan Lu, Yajun Wang, Zhen Zhang, Jingliang Huang, Meicun Yao, Guobin Huang, Yuanyuan Ge, Peichun Zhang, Huaxin Huang, Yong Wang, Huiliang Li, Wen Wang

**Affiliations:** ^1^Huamin Medicine Co Ltd., Zhuhai, China; ^2^Wolfson Institute for Biomedical Research, University College London, London, UK; ^3^Joint Center for Life Sciences, School of Life Sciences, Tsinghua University, Beijing, China; ^4^School of Chemical Engineering and Technology, Sun Yat-sen University, Zhuhai, China; ^5^School of Pharmaceutical Sciences, Sun Yat-sen University, Shenzhen, China; ^6^Levi Regenerative Medicine Technologies, Zhuhai, China; ^7^Guangzhou Hongxiang Biological Medicine Technology Co Ltd., Guangzhou, China

## Abstract

This new decade has started with a global pandemic of COVID-19 caused by severe acute respiratory syndrome coronavirus 2 (SARS-CoV-2), precipitating a worldwide health crisis and economic downturn. Scientists and clinicians have been racing against time to find therapies for COVID-19. Repurposing approved drugs, developing vaccines and employing passive immunization are three major therapeutic approaches to fighting COVID-19. Chicken immunoglobulin Y (IgY) has the potential to be used as neutralizing antibody against respiratory infections, and its advantages include high avidity, low risk of adverse immune responses, and easy local delivery by intranasal administration. In this study, we raised antibody against the spike (S) protein of SARS-CoV-2 in chickens and extracted IgY (called IgY-S) from egg yolk. IgY-S exhibited high immunoreactivity against SARS-CoV-2 S, and by epitope mapping, we found five linear epitopes of IgY-S in SARS-CoV-2 S, two of which are cross-reactive with SARS-CoV S. Notably, epitope SIIAYTMSL, one of the identified epitopes, partially overlaps the S1/S2 cleavage region in SARS-CoV-2 S and is located on the surface of S trimer in 3D structure, close to the S1/S2 cleavage site. Thus, antibody binding at this location could physically block the access of proteolytic enzymes to S1/S2 cleavage site and thereby impede S1/S2 proteolytic cleavage, which is crucial to subsequent virus-cell membrane fusion and viral cell entry. Therefore, the feasibility of using IgY-S or epitope SIIAYTMS-specific IgY as neutralizing antibody for preventing or treating SARS-CoV-2 infection is worth exploring.

## 1. Introduction

Following the first reported cases of unexplained pneumonia in December 2019 in Wuhan, China [1, 2], severe acute respiratory syndrome coronavirus 2 (SARS-CoV-2) has since been confirmed to be the pathogen of a novel infectious respiratory disease, namely, coronavirus disease 2019 (COVID-19) [3–5]. In March 2020, the World Health Organization (WHO) declared COVID-19 a global pandemic [6]. At present, the counts of COVID-19 stand at above 37 million confirmed cases including over 1 million recorded deaths worldwide. As COVID-19 continues to rage in some parts of the world and threatens new waves of infection in others with devastating consequences for people's lives and livelihoods as well as global economy [7], all-round scientific effort towards effective disease management and treatment is urgently needed. Repurposing approved drugs and developing specific vaccines are two main strategies to combat SARS-CoV-2 infection. So far, several repurposed drugs such as remdesivir, an adenosine nucleoside triphosphate analog previously tested for treating Ebola virus disease [8, 9], and chloroquine/hydroxychloroquine, a commonly used antimalaria drug, have produced unsatisfactory results in several COVID-19 clinical trials [10–12]. Despite the fast progress on developing vaccines for SARS-CoV-2 [13–15], we are still faced with uncertainty about the effectiveness and mass production of COVID-19 vaccines [16]. Passive immunization by introducing pregenerated antibodies/immunoglobulins is another old-fashioned treatment being eyed with renewed interest for fighting COVID-19, particularly for patients with immunodeficient conditions [17]. For example, convalescent plasma transfusion has been shown to help improve the clinical outcome of severe COVID-19 [18, 19], but issues regarding supply, safety, and clinical efficacy need to be further addressed in randomized controlled trials [20]. Moreover, several human monoclonal antibodies could neutralize SARS-CoV-2 and inhibit its infectious ability in cultured systems [21–24]. Apart from human antibodies, chicken immunoglobulin Y (IgY) from egg yolk has proved able to neutralize pathogens in the respiratory tract of mice [25–27]. Because of its high specificity and avidity, low risk of adverse immune responses, low manufacture cost, and ease of storage, chicken IgY raised against SARS-CoV-2 is waiting to be tapped into for potential therapeutic application in treating COVID-19 [28, 29].

SARS-CoV-2 belongs to the coronavirus family, which is a large family of enveloped, single-stranded positive-sense RNA viruses, comprised of alpha, beta, gamma, and delta four subgroups [30]. To date, seven coronaviruses have been identified as being able to infect human and four of them (HCoV-NL63, HCoV-229E, HCoV-OC43, and HCoV-HKU1) have been linked to mild colds, whereas the other three [Middle East respiratory syndrome coronavirus (MERS-CoV), SARS-CoV, and SARS-CoV-2] can lead to severe respiratory infection. MERS-CoV, SARS-CoV, SARS-CoV-2, HCoV-OC43, and HCoV-HKU1 are members of the beta subgroup of coronaviruses. Coronavirus genome encodes four structural proteins, i.e., the spike (S), envelope (E), membrane (M), and nucleocapsid (N) proteins [31]. The highly glycosylated homotrimeric S protein can be cleaved into two subunits, S1 and S2, via host-dependent proteolytic cleavage; the S1 subunit contains a receptor-binding domain (RBD), mediating host receptor recognition, while the S2 subunit anchors the spike in the viral envelope, facilitating virus-cell membrane fusion and viral cell entry [32–34]. Upon interaction between the S1 subunit and its host receptor, conformational changes trigger further cleavage of S2 subunit at the S2′ site located immediately upstream of the fusion peptide, the exposure of which leads to membrane fusion and virus invasion [34]. The SARS-CoV S and SARS-CoV-2 S share human angiotensin-converting enzyme 2 (hACE2) as receptor, whereas the MERS S uses dipeptidyl peptidase 4 (DPP4) as receptor. Targeting SARS-CoV-2 S with antibody to block virus-host interaction and thus prevent viral invasion is generally the rationale for vaccine design and for the passive immunization approach [13, 14]. In this study, we immunized chickens with recombinant S protein of SARS-CoV-2 and acquired egg yolk IgY (called IgY-S) with high immunoreactivity. By epitope mapping, we further identified five epitopes of IgY-S in SARS-CoV-2 S, two of which are cross-reactive between SARS-CoV-2 S and SARS-CoV S. Our results lay the foundation for further study on IgY-S as a potential treatment for preventing or combating SARS-CoV-2 infection.

## 2. Materials and Methods

### 2.1. IgY-S Generation in Hens

Full-length extracellular domain of SARS-CoV-2 S protein (1-1,213 amino acids; GenBank No. YP_009724390), fused with a polyhistidine tag at the C-terminus, was expressed with the baculovirus-insect cell expression system (Sino Biological, Beijing). 100 *μ*g purified protein was emulsified with an equal volume of Freund's complete adjuvant (Sigma-Aldrich, MO) and intramuscularly injected into the thigh of laying hens at 5 sites. Two booster injections of 50 *μ*g purified protein mixed with an equal volume of incomplete Freund's adjuvant were administered at two-week intervals. One week after the final injection, eggs were collected and stored at 4°C. Hens injected with only adjuvant in parallel were used for acquiring control IgY (IgY-C).

### 2.2. IgY Isolation

The egg yolk was carefully separated from the white, rinsed twice with phosphate-buffered saline (PBS), transferred into an equal volume of 5.33% PEG6000 solution, mixed thoroughly for 15 minutes, and centrifuged at 7,000 × g for 7 minutes at 4°C. The supernatant was carefully transferred, and an equal volume of 40% PEG6000 was slowly added to it. After thorough mixing for 15 minutes, the mixture was centrifuged at 7,000 × g for 7 minutes at 4°C. After removal of the supernatant, the pellet was dissolved into 10 ml PBS with vortexing, followed by centrifugation at 7,000 × g for 7 minutes at 4°C; then, the supernatant was collected and filtered with a 0.22 *μ*m filter. The extracted IgY concentration was determined by the Bradford Reagent (Bio-Rad Laboratories, Shanghai). IgY was freeze-dried for storage at −20°C or stored at 1 mg/ml at −20°C.

### 2.3. Enzyme-Linked Immunosorbent Assay (ELISA)

MaxiSorb 96-well plates (Thermo Fisher Scientific, Guangzhou) were coated with 100 *μ*l per well of the recombinant extracellular S protein of SARS-CoV-2 at 1.5 *μ*g/ml for 2 hours at 37°C, washed 3 times, and saturated with blocking buffer (5% nonfat dried milk in PBS) for 2 hours at 37°C. IgY stock solution (1 mg/ml) was diluted in blocking buffer to make 1 : 500 dilution, followed by a two-fold serial dilution. The plates were washed, and then 100 *μ*l of serially diluted IgY-S or IgY-C was added in triplicate to the wells and incubated for 1.5 hours at 37°C. The plates were washed and incubated with 100 *μ*l per well of 1 : 2,000 HRP-conjugated goat anti-chicken IgY (Santa Cruz Biotechnology, CA) for 1 hour at 37°C. After washing, 100 *μ*l TMB substrate solution (Hongxiang Biotech, Guangzhou) was added to the wells and incubated for 3 minutes at 37°C; then, the reaction was terminated with 50 *μ*l of 2M H_2_SO_4_. The plates were scanned on a multiwell spectrophotometer, and optical density was read at 450 nm.

### 2.4. Epitope Mapping

IgY-S epitope mapping was performed using the PEPperPRINT peptide microarray system (PEPperPRINT GmbH, Heidelberg). IgY-C was used as mapping control. Microarray assays were conducted using the SARS-CoV-2 Proteome Microarray made up by duplicated spots of 4,883 different peptides, the SARS-CoV Antigen Microarray composed of duplicated spots of 998 different peptides, and the MERS-CoV Proteome Microarray consisting of duplicated spots of 3,818 different peptides. Hemagglutinin (HA) peptides framing the peptide microarrays were used as internal quality control to monitor assay quality and amino acid integrity. Briefly, to investigate background interactions, microarrays were prestained with 0.2 *μ*g/ml DyLight680-conjugated goat anti-chicken IgY (H+L) secondary antibody (Thermo Fisher Scientific) and 0.5 *μ*g/ml DyLight800-conjugated mouse monoclonal anti-HA (12CA5) control antibody (Thermo Fisher Scientific) in incubation buffer at room temperature for 45 minutes. Subsequently, sets of microarrays were incubated with IgY-S and IgY-C at concentrations of 1 *μ*g/ml and 10 *μ*g/ml in incubation buffer for 16 hours at 4°C with shaking at 140 rpm. After washing, the microarrays were incubated with goat anti-chicken secondary antibody and mouse anti-HA control antibody in incubation buffer for 45 minutes at room temperature. Stained microarrays were scanned with the LI-COR Odyssey imaging system (Li-cor Biosciences, Bad Homburg) with parameters set as: scanning offset 0.65 mm, resolution 21 *μ*m and scanning intensities 7/7 (red = 680 nm/green = 800 nm). Microarray image analysis was conducted with PepSlide Analyzer (SICASYS Software, Germersheim). A software algorithm was used to break down the fluorescence intensity of each spot into raw, foreground, and background signals and to calculate average median foreground intensities and spot-to-spot deviations of duplicated spots. Based on average median foreground intensities, intensity maps were generated and incorporated in the peptide maps. Peptide spots related to SARS-CoV-2 S as well as SARS-CoV S were focused on for analysis.

## 3. Results

### 3.1. IgY-S Immunoreactivity

**Figure 1:**
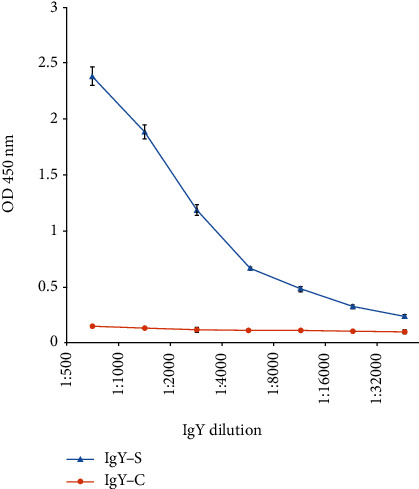
ELISA assay of IgY-S immunoreactivity. ELISA graph showing IgY-S immunoreactivity against SARS-CoV-2 S. Plates coated with recombinant extracellular S protein of SARS-COV-2 were incubated with IgY-S or IgY-C (control) at a series of dilutions (1 mg/ml of stock concentration for both antibodies), and optical density was read at 450 nm (OD 450 nm). The results were plotted as OD 450 nm readings versus IgY dilutions. Mean ± SD (*n* = 3) are presented.

IgY-S isolated from egg yolk of immunized chickens was evaluated for reactivity with recombinant S protein of SARS-CoV-2 by ELISA. Compared to IgY-C control, IgY-S displayed significantly elevated ability to bind to the antigen, even at the greatest dilution (1: 32,000)/lowest concentration (0.03125 *μ*g/ml) tested (*p* = 0.008, Student's *t-*test) (Figure 1). These data hint at high immunoreactivity of IgY-S against SARS-CoV-2 S.

### 3.2. IgY-S Epitopes in SARS-CoV-2 S

**Figure 2:**
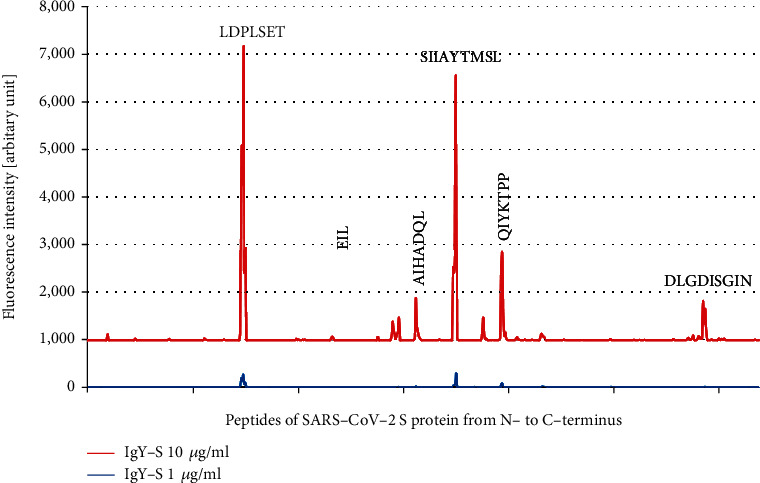
IgY-S epitope mapping against the SARS-CoV-2 Proteome Microarray. Peptide microarrays were performed with IgY-S at 1 *μ*g/ml and 10 *μ*g/ml, and stained microarrays were scanned, and signals were collected. Fluorescence intensities of peptide spots were generated with the PepSlide Analyzer software and plotted against sequential peptides covering SARS-CoV-2 S protein from N- to C-terminus. Fluorescence intensity peaks of peptides containing the consensus motifs LDPLSET, SIIAYTMSL, QIYKTPP, AIHADQL, DLGDISGIN, and EIL are indicated. Weak signal peaks from peptide spots not of “epitope signal pattern” are also shown.

**Figure 3:**
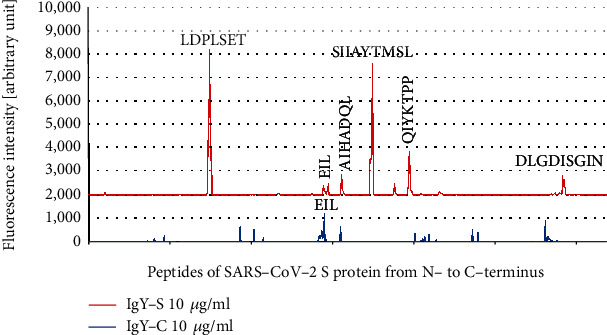
Signal analysis of IgY-C against the SARS-CoV-2 Proteome Microarray. A peptide microarray was performed with IgY-C (10 *μ*g/ml), and the resulting fluorescence intensities were plotted against sequential peptides covering SARS-CoV-2 S protein from N- to C-terminus. The fluorescence intensity plot of IgY-S (10 *μ*g/ml) was incorporated for comparison. Weak signal peaks from peptide spots not of “epitope signal pattern” can be seen, and some of these peptides contain an N-terminal TD motif. Signal peaks corresponding to motif EIL are present for both IgY-S and IgY-C.

IgY-S epitope mapping against SARS-CoV-2 S was performed using the SARS-CoV-2 Proteome Microarray made up by 15 amino acid peptides of SARS-CoV-2 with peptide-peptide overlaps of 13 amino acids. The microarrays were incubated with IgY-S at 1 *μ*g/ml and 10 *μ*g/ml. Prestaining of the microarray with the secondary antibody and anti-HA control antibody did not highlight any background interaction with SARS-CoV-2 peptides that could have interfered with the main assay. As standard epitopes are normally shorter than 12 amino acids with the majority of the binding energy typically being derived from around 5 amino acids [35], an epitope should be encompassed by a series of contiguous overlapping peptides and displayed as a distinct signal formation of successive spots (i.e., an “epitope signal pattern”). Six clear such patterns were observed, corresponding to consensus sequence motifs LDPLSET, SIIAYTMSL, QIYKTPP, AIHADQL, DLGDISGIN, and EIL, in order of signal strength from high to low (Figure 2). However, an “epitope signal pattern” was also traced back to sequence motif EIL when IgY-C was used instead of IgY-S (Figure 3), suggesting nonspecific binding of chicken IgY to this motif. In addition, signals from a number of single peptides containing an N-terminal motif TD were detected with IgY-C, but they were rather atypical, not of “epitope signal pattern.” Additional signals were detected in the microarray regions covering other SARS-CoV-2 proteins such as N protein and Orf1ab, but most were weak, possibly caused by nonspecific interaction or noise (data not shown). Taken together, these data reveal five linear motifs, LDPLSET, SIIAYTMSL, QIYKTPP, AIHADQL, and DLGDISGIN 5, as epitopes of IgY-S in SARS-CoV-2 S.

### 3.3. IgY-S Cross-Reactivity with SARS-CoV S

**Figure 4:**
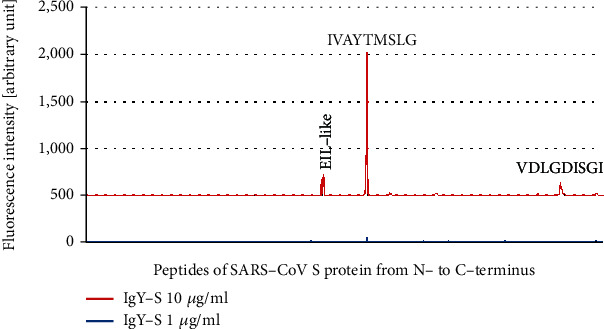
IgY-S epitope mapping against the SARS-CoV Antigen Microarray. Peptide microarrays were performed with IgY-S at 1 *μ*g/ml and 10 *μ*g/ml, and the resulting fluorescence intensities were plotted in relation to sequential peptides covering SARS-CoV-2 S protein from N- to C-terminus. Fluorescence intensity peaks corresponding to the consensus motifs IVAYTMSLG and VDLGDISGI as well as an EIL-like motif are indicated. Weak signal peaks from peptide spots not of “epitope signal pattern” are also shown.

IgY-S epitope mapping against SARS-CoV S protein was performed using the SARS-CoV Antigen Microarray. Three relatively weak but clear “epitope signal patterns” were observed, two of them corresponding to consensus sequence motifs IVAYTMSLG and VDLGDISGI (Figure 4). These two epitopes in SARS-CoV S, IVAYTMSLG and VDLGDISGI, share high sequence homology with epitopes SIIAYTMSL and DLGDISGIN in SARS-CoV-2 S, respectively (identical/similar amino acids underlined). The other sequence motif recognized by IgY-S was an EIL-like epitope, but again the recognition was repeated when IgY-C was used (data not shown) pointing to nonspecific interaction.

Taken all, the above data demonstrate that IgY-S can cross-react with SARS-CoV S via two cross-reactive epitopes between SARS-CoV-2 S and SARS-CoV S, but its epitopes QIYKTPP and AIHADQL appear specific to SARS-CoV-2 S. In addition, IgY-S did not show cross-reactivity with MERS S in a peptide microarray assay using the MERS-CoV Proteome Microarray (data not shown).

### 3.4. IgY-S Epitope Locations in SARS-CoV-2 S

**Figure 5:**
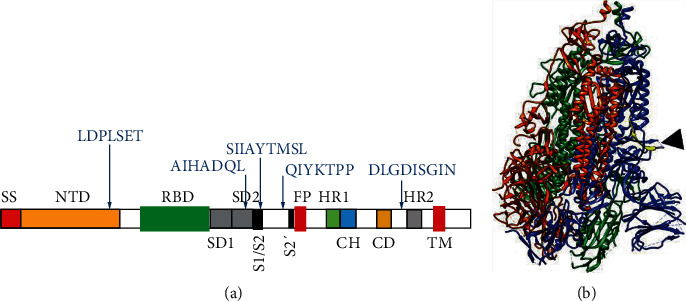
Schematic illustrations of SARS-Cov-2 S protein. (a) Locations of IgY-S epitopes in SARS-Cov-2 S. Locations of the five identified IgY-S epitopes are indicated by arrows. SS: signal sequence; NTD: N-terminal domain; RBD: receptor-binding domain; SD1: subdomain 1; SD2: subdomain 2; S1/S2: S1/S2 cleavage region; S2′: S2′ cleavage region; FP: fusion peptide; HR1: heptad repeat 1; CH: central helix; CD: connector domain; HR2: heptad repeat 2. (b) 3D view of SARS-CoV-2 S trimer based on PDB 6VXX [37], constructed by UCSF Chimera. The S1/S2 cleavage site is indicated by an arrowhead. The yellow strand represents a peptide containing epitope SIIAYTMSL.

Similar to SARS-CoV S, the extracellular region of SARS-CoV-2 S contains from N- to C-terminus, signal sequence (SS), N-terminal domain (NTD), RBD, subdomains 1 and 2 (SD1 and SD2), S1/S2 cleavage region, S2′ cleavage region, fusion peptide (FP), heptad repeat 1 (HR1), central helix (CH), connector domain (CD), and heptad repeat 2 (HR2) (Figure 5) [36, 37]. Epitope LDPLSET is located at the C-terminus of NTD; SIIAYTMSL partially overlaps S1/S2 cleavage region; QIYKTPP is located between S1/S2 cleavage region and S2′ cleavage region; AIHADQL lies within SD2; and DLGDISGIN resides between CD and HR2. In epitope SIIAYTMSL, the SI residues overlapping the S1/S2 cleavage region are thought to be conserved for SARS-CoV-2 and SARS-CoV and may have a bearing on S1/S2 cleavage [37]. In addition, it is possible that antibody binding at this location could interfere with S1/S2 proteolytic cleavage, thereby inhibiting membrane fusion and viral entry into the host cell [38].

## 4. Discussion

Passive immunization is an important approach to tackling SARS-CoV-2 infection [39]. In this study, we raised antibody in chickens against recombinant extracellular S protein of SARS-CoV-2 and obtained egg yolk IgY-S with high immunoreactivity. By epitope mapping, we identified five linear epitopes of IgY-S in SARS-CoV-2 S protein (Figures 2 and 3), two of which are cross-reactive with SARS-CoV S (Figure 4).

The SIIAYTMSL epitope, which showed the second highest fluorescence intensity in peptide microarray analysis, is a conserved sequence between SARS-CoV S and SARS-CoV-2 S and overlaps the S1/S2 cleavage region in both S proteins. With the help of the published 3D structure of SARS-CoV-2 S [37], we found that this epitope is located on the surface of S homotrimer, close to the S1/S2 cleavage site (Figure 5), hinting that antibody binding at this location could physically block the access of proteolytic enzymes to the cleavage site. Given the importance of S1/S2 proteolytic cleavage to subsequent membrane fusion and viral cell invasion, it is possible that IgY-S binding to epitope SIIAYTMSL could prevent virus infection [38]. The spatial locations of the other four epitopes are either inside S trimer or difficult to identify with current resolution for the 3D structure. Nevertheless, neutralizing experiments in cultured human cells have been planned to evaluate whether to use IgY-S as a whole or to acquire epitope-specific IgY by affinity purification with each epitope.

As egg yolk IgY can be produced quickly (5 weeks of immunization period) with high yield (40-80 mg per egg) [40], neutralizing IgY has unique advantages as a potential passive immunization therapy for infections by new pathogens, e.g., SARS-CoV-2 with a new mutated form of S protein, which is possible because RNA viruses are known for their high mutation rates [41]. An easy and noninvasive way to deliver agents locally for respiratory tract infection is by intranasal or oral administration. In animal models, egg yolk IgY isolated from immunized chickens has been tested for treating viral respiratory infections by influenza A subtypes H1N1, H3N2, and H5N1 [25–27] and influenza B [42] via intranasal delivery. As chicken eggs are a dietary staple for human, IgY is well tolerated in human [28]. Moreover, because purified IgY does not contain the allergenic egg albumin, it can also be safely used by patients with egg allergy [28]. In a double-blind, randomized, placebo-controlled trial, a specific IgY antibody was effective in treating patients with acute and chronic pharyngitis when administered by oral spray [43], suggesting that IgY could be a safe and effective agent against respiratory infection. Therefore, our IgY-S has the potential to be safely used as a nasal or oral spray to prevent/treat SARS-CoV-2 infection [44]. If our planned in vitro neutralizing assays produce positive results, we will be encouraged to conduct an early-phase clinical trial with IgY-S. In addition, IgY does not interact with mammalian Fc receptors or activate the mammalian complement system, so it can avoid triggering antibody-dependent enhancement (ADE) of disease and complement-mediated adverse inflammatory responses [45]. Therefore, it could be beneficial to administer neutralizing IgY intravascularly to treat SARS-CoV-2 infection, especially to patients with severe symptoms.

Although the great potential of chicken IgY for COVID-19 treatment has been proposed by several labs [29, 44, 46], so far, no follow-up data or epitopes of SARS-CoV-2 targeted by IgY have been published. A previous study on chicken IgY produced against SARS-CoV implied that a dominant epitope existed in the “Se-e” fragment located between amino acid residues 456–650 of SARS-CoV S [47]. The AIHADQL epitope identified in our study is located in the counterpart of “Se-e” fragment in SARS-CoV-2 S. Although AIHADQL is found in “Se-e” fragment of SARS-CoV, it was not recognized by IgY-S in the SARS-CoV peptide microarray assay. The reason could lie in the neighbouring amino acids, which are dissimilar between SARS-CoV and SARS-CoV-2 and may play a role in antibody-epitope binding. In addition, continued search for neutralizing epitopes/antibodies has been conducted using convalescent sera of COVID-19 patients, with the focus on RBD of SARS-CoV-2 S protein [23, 24, 48–52]. Neutralizing epitopes in other domains of S1 subunit such as NTD, as well as domains of S2 subunit, have also been reported [53–56]. However, none of these epitopes are identical to epitopes identified by IgY-S. Interestingly, peptides/epitopes in RBD were found to have less interaction with convalescent sera in a SARS-CoV-2 peptide screening study [57]. The reason behind this could be that RBD epitopes are conformational epitopes or glycosylated, and this is a common limitation to most epitope mapping analyses, ours included.

## 5. Conclusions

In this study, chicken IgY-S was generated with high immunoreactivity against SARS-CoV-2 S protein and five epitopes of IgY-S were identified. Our work provides a new potential therapeutic tool for COVID-19 treatment. Our study is the first to explore using chicken egg IgY for neutralizing SARS-CoV-2.

## Data Availability

The epitope microarray data used to support the findings of this study are available from the corresponding author upon request.
